# Screening Antitumor Bioactive Fraction from *Sauromatum giganteum* (Engl.) *Cusimano & Hett* and Sensitive Cell Lines with the Serum Pharmacology Method and Identification by UPLC-TOF-MS

**DOI:** 10.3390/molecules20034290

**Published:** 2015-03-06

**Authors:** Shi-Yong Gao, Yun-Fei Gong, Qiu-Jia Sun, Jing Bai, Long Wang, Zi-Quan Fan, Yu Sun, Yi-Jun Su, Jian Gang, Yu-Bin Ji

**Affiliations:** 1 The Institute of Materia Medica, The Research Center of Life Sciences and Environmental Sciences, Harbin Commerce University, Harbin 150076, Heilongjiang, China; E-Mails: gyf.128@163.com (Y.-F.G.); sqj0227@sohu.com (Q.-J.S.); baijing0308@163.com (J.B.); wlharbin@163.com (L.W.); sunyujxyz@163.com (Y.S.); suyijunharbin@163.com (Y.-J.S.); gangjian1986@163.com (J.G.); jyb@hrbcu.edu.cn (Y.-B.J.); 2 Engineering Research Center of Natural Anticancer Drugs of Ministry of Education, Harbin University of Commerce, Harbin 150076, Heilongjiang, China; 3 China Solution Center, Waters Technolygies (Shanghai) Ltd., Shanghai 201206, China; E-Mail: ZiQuan_Fan@waters.com

**Keywords:** *Sauromatum giganteum* (Engl.) *Cusimano & Hett*, bioactive solvent fraction, sensitive cell lines, serum pharmacology method, UPLC-TOF-MS

## Abstract

*Sauromatum giganteum* (Engl.) *Cusimano & Hett* Tuber are used in Chinese folklore medicine for treatment of neoplasms. However, the claim has not been scientifically validated. The aim of the study is to screen the antitumor bioactive fraction of *Sauromatum giganteum* (Engl.) *Cusimano & Hett* Tuber and sensitive tumor cell lines using a cytotoxicity assay *in vitro* and tumor transplantation method *in vivo*, to support its use in folk medicine. The petroleum ether fraction, chloroform fraction, ethyl acetate fraction, *n*-butanol fraction and water fraction were successively extracted by turn by the maceration under reflux assay. Screening of antitumor bioactive fraction and sensitive cell lines were measured by MTT assay and the serum pharmacology method, and *in vivo* the antitumor activities of the active fraction was evaluated by using S_180_ or H_22_ tumor-bearing mice model and Kunming mice. The active constituents of ethyl acetate fraction of *Sauromatum giganteum* (Engl.) *Cusimano & Hett* were characterized by UPLC-TOF-MS. Compared with control groups, mice serum containing ethyl acetate fraction had a inhibition effect on SMMC-7721 cell, SGC-7901 cell, MCF-7 cell, HeLa cell, A549 cell, HT-29, and MDA-MB-231, respectively, but mice serum containing other four fractions had no different with that of control group. The inhibition capabilities of mice serum containing ethyl acetate fraction on the seven cell lines in descending order is SGC-7901 > SMMC-7721 > MCF-7 > HT-29 > A549 > HeLa > MDA-MB-231. *In vivo* the inhibition rate of 106, 318, 954 mg/kg·d ethyl acetate fraction dry extract to sarcoma S_180_ is 15.22%, 26.15% and 40.24%, respectively, and life prolonging rate to hepatoma H_22_ is 33.61%, 40.16% and 55.74%. A total of 14 compounds were identified in the ethyl acetate fraction of *Sauromatum giganteum* (Engl.) *Cusimano & Hett.* The results of the experimental studies proved the antitumor activity of *Sauromatum giganteum* (Engl.) *Cusimano & Hett* and supported the traditional use of this plant. These data indicate the potential for the use of ethyl acetate fraction of *Sauromatum giganteum* (Engl.) *Cusimano & Hett* Tuber in tumor therapy, anti-tumor activity on cancer cell line in descending order is SGC-7901 > SMMC-7721 > MCF-7 > HT-29 > A549 > HeLa > MDA-MB-231.

## 1. Introduction

Cancer is the leading cause of death in economically developed countries and the second leading cause of death in developing countries. In recent years, the increase in the number of cancer cases has motivated the growth of cancer research [1]. A large number of natural products and dietary components have been evaluated as potential chemopreventive agents, and herbal remedies used in traditional folk medicine provide a largely unexplored source of potential novel drugs [2]. The traditional Chinese medicine have a long time clinical practice, so it is a very safe and effective cure [3].

*Sauromatum giganteum* (Engl.) *Cusimano & Hett* is a herbal plant, a synonym is *Typhonium giganteum* Engl. or *Typhonium giraldii* (Baroni) Engl. or *Typhonium stoliczkae* Engl., the dried root tuber of which is named Baifuzi in Chinese and recorded in Chinese pharmacopoeia as a traditional Chinese medicine [4,5]. It has the effect of “dispelling wind-phelgm”, and been used for the folklore treatment of cancer for a long time in Northeast of China.

There are a few research on *Sauromatum giganteum* (Engl.) *Cusimano & Hett*, Chen XS *et al.* reported the chemical components of *Typhonium giganteum* Engl. tuber [6] and synthesis method of a new cerebroside isolated from *Typhonium giganteum* Engl [7,8], and Chi S reported that Baifuzi reduces transient ischemic brain damage through an interaction with the STREX domain of BKCa channels [4], and the cerebrosides [9] from baifuzi, a novel potential blocker of calcium-activated chloride channels in rat pulmonary artery smooth muscle cells [5], is the active compounds of activation of BKCa channels [10].

As concerns experimental research on the anti-tumor activity, only Li Q *et al.* reported that SFE-CO_2_ extract from *Typhonium giganteum* Engl. Tubers induced apoptosis in human hepatoma SMMC-7721 cells [11]. Ma YL reported the up-regulation of TRAIL/TRAIL-R1 and TRAIL-R2 by Lignans of Rhizoma Typhonii could be involved in the induction of apoptosis in SGC-7901 cells [12]. Shan BE found Rhizoma typhonii extract has immunoenhancing activity to human T cell and macrophage, through stimulating the killer cell and phagocytosis of tumor cell and allo-antigen, which could be used clinically for modulating immune responses and for treating tumor and other diseases [13]. Wang SQ reported the effect of aqueous extract of *Typhonium giganteum* on genes expression in hepatocellular carcinoma SMMC-7721 cells [14].

Study of traditional Chinese medicine is very difficult because the chemical components are very complex. “Serum pharmacology” was first presented by Tashino [15], a Japanese scholar, in 1984. The theory stated that only chemical components of a Chinese herb absorbed in blood could exert their activity on diseases [16]; therefore, the serum pharmacology method provides a good research approach for traditional Chinese medicine [17,18,19,20], which could avoid the interference of the chemical components not absorbed in blood [21,22]. This method not only reveals the mechanism of the active ingredient’s action due to its excellent controllability of experimental conditions and detection convenience, but also prevents the interference from crude herbal, so it is more scientific and real. Until now, serum-pharmacology has been widely utilized to explore herbal or traditional Chinese formulations because of its advantage. In addition, it has been widely applied in the research of natural medicine in China [23,24,25,26] and East Asian countries in recent years [27].

The antitumor bioactive solvent fraction of *Sauromatum giganteum* (Engl.) *Cusimano & Hett* Tuber and cytotoxicity on different tumor cell lines has not been revealed. In this paper, the serum pharmacology method and MTT assay *in vitro* and tumor transplantation method *in vivo* were adopted to determine the antitumor bioactive fraction of *Sauromatum giganteum* (Engl.) *Cusimano & Hett* tuber, sensitive cell lines and antitumor activity *in vivo*. The results might provide a scientific explanation for the folk or traditional application of *Sauromatum giganteum* (Engl.) *Cusimano & Hett* in cancer therapy.

## 2. Results and Discussion

### 2.1. Antiproliferative Effect of Fraction-Containing Serum on 7 Human Tumor Cell Lines

The effects of the five different fraction-containing serums (petroleum ether fraction, chloroform fraction, ethyl acetate fraction, *n*-butanol fraction, water fraction) on the proliferation of SMMC-7721, SGC-7901, MCF-7, HeLa, A549, HT-29, and MDA-MB-231 cell lines assessed using the MTT assay are shown in Table 1, Table 2, Table 3, Table 4, Table 5, Table 6, Table 7 and Table 8. Compared with the control group (negative control of fetal bovine serum, negative control of mice serum containing water or negative control of mice serum containing olive oil), treated cells with serum containing ethyl acetate fraction had a decline in viability. The inhibition rate was 71.31% on SMMC-7721 cell, 73.89% on SGC-7901 cell, 50.75% on MCF-7 cell, 22.75% on HeLa cell, 23.05% on A549 cell, 34.81% on HT-29, and 12.28% on MDA-MB-231, respectively. Viability of other fraction groups had no different with that of control group. The positive control ADR or mice serum containing 5-FU was very toxic with a significant inhibition rate.

**Table 1:** Effects of mice serum containing fractions of *Sauromatum giganteum* (Engl.) *Cusimano & Hett* Tuber on proliferation of SMMC-7721 cell.

Groups	Dose (μg·mL^−1^)	Oral Administration Dose (g·kg^−1^·d^−1^)	Serum Additive Volume (%)	OD Value	Inhibition Rate (%)
control-FBS	—	—	10	1.0200 ± 0.0305	—
water-control-S	—	—	10	1.0225 ± 0.0601	—
oil-control-S	—	—	10	1.0223 ± 0.0509	—
ADR	0.065	—	10	0.5352 ± 0.0309 **	47.53
5-FU-S	—	0.1000	10	0.3265 ± 0.0096 ^∆∆^	74.21
PEF-S	—	1.1500	10	0.9920 ± 0.0587	2.97
CF-S	—	1.0140	10	1.0032 ± 0.0492	1.87
EAF-S	—	0.9540	10	0.2933 ± 0.0295 ^##^	71.31
BF-S	—	4.6350	10	1.0722 ± 0.0752	4.86
WF-S	—	4.7575	10	1.0270 ± 0.0386	0.44

Note: ** *p* < 0.01 compared with Negative control of fetal bovine serum (control-FBS); ^##^ *p* < 0.01 compared with negative control mice serum containing olive oil (oil-control-S); ^∆∆^ *p* < 0.01 compared with negative control mice serum containing water (water-control-S).

**Table 2:** Effects of mice serum containing fractions of *Sauromatum giganteum* (Engl.) *Cusimano & Hett* Tuber on proliferation of SGC-7901 cell (*n* = 6).

Groups	Dose (μg·mL^−1^)	Oral Administration Dose (g·kg^−1^·d^−1^)	Serum Additive Volume (%)	OD Value	Inhibition Rate (%)
control-FBS	—	—	10	0.8082 ± 0.0104	—
water-control-S	—	—	10	0.8052 ± 0.0115	—
oil-control-S	—	—	10	0.8080 ± 0.0151	—
ADR	1.03	—	10	0.4092 ± 0.0064 **	50.62
5-FU-S	—	0.1000	10	0.2823 ± 0.0118 ^∆∆^	64.97
PEF-S	—	1.1500	10	0.7993 ± 0.0081	1.11
CF-S	—	1.0140	10	0.7752 ± 0.0119	4.08
EAF-S	—	0.9540	10	0.2113 ± 0.0093 ^##^	73.89
BF-S	—	4.6350	10	0.7647 ± 0.0177	4.97
WF-S	—	4.7575	10	0.7890 ± 0.0093	1.99

Note: ** *p* < 0.01 compared with Negative control of fetal bovine serum (control-FBS); ^##^ *p* < 0.01 compared with negative control mice serum containing olive oil (oil-control-S); ^∆∆^ *p* < 0.01 compared with negative control mice serum containing water (water-control-S).

**Table 3:** Effects of mice serum containing fractions of *Sauromatum giganteum* (Engl.) *Cusimano & Hett* Tuber on proliferation of MCF-7 cell (*n* = 6).

Groups	Dose (μg·mL^−1^)	Oral Administration Dose (g·kg^−1^·d^−1^)	Serum Additive Volume (%)	OD Value	Inhibition Rate (%)
control-FBS	—	—	10	1.0578 ± 0.0178	0
water-control-S	—	—	10	1.0758 ± 0.0185	0
oil-control-S	—	—	10	1.0498 ± 0.0160	0
ADR	0.148	—	10	0.5160 ± 0.0166 **	51.23
5-FU-S	—	0.1000	10	0.6527 ± 0.0253 ^∆∆^	39.31
PEF-S	—	1.1500	10	1.0200 ± 0.0271	2.84
CF-S	—	1.0140	10	0.9765 ± 0.0315	7.66
EAF-S	—	0.9540	10	0.5172 ± 0.0226 ^##^	50.75
BF-S	—	4.6350	10	0.9905 ± 0.0193	7.90
WF-S	—	4.7575	10	1.0665 ± 0.0189	0.84

Note: ** *p* < 0.01 compared with Negative control of fetal bovine serum (control-FBS); ^##^ *p* < 0.01 compared with negative control mice serum containing olive oil (oil-control-S); ^∆∆^ *p* < 0.01 compared with negative control mice serum containing water (water-control-S).

**Table 4:** Effects of mice serum containing fractions of *Sauromatum giganteum* (Engl.) *Cusimano & Hett* Tuber on proliferation of HeLa cell (*n* = 6).

Groups	Dose (μg·mL^−1^)	Oral Administration Dose (g·kg^−1^·d^−1^)	Serum Additive Volume (%)	OD Value	Inhibition Rate (%)
control-FBS	—	—	10	0.9957 ± 0.0144	0
water-control-S	—	—	10	0.9780 ± 0.0042	0
oil-control-S	—	—	10	0.9763 ± 0.0108	0
ADR	0.362	—	10	0.4972 ± 0.0055 **	50.10
5-FU-S	—	0.1000	10	0.5910 ± 0.0054 ^∆∆^	38.55
PEF-S	—	1.1500	10	0.9628 ± 0.0100	1.33
CF-S	—	1.0140	10	0.9442 ± 0.0047	3.28
EAF-S	—	0.9540	10	0.7540 ± 0.0073 ^##^	22.75
BF-S	—	4.6350	10	0.9503 ± 0.0057	2.86
WF-S	—	4.7575	10	0.9667 ± 0.0055	1.12

Note: ** *p* < 0.01 compared with Negative control of fetal bovine serum (control-FBS); ^##^ *p* < 0.01 compared with negative control mice serum containing olive oil (oil-control-S); ^∆∆^ *p* < 0.01 compared with negative control mice serum containing water (water-control-S).

**Table 5:** Effects of mice serum containing fractions of *Sauromatum giganteum* (Engl.) *Cusimano & Hett* Tuber on proliferation of A549 cell (*n* = 6).

Groups	Dose (μg·mL^−1^)	Oral Administration Dose (g·kg^−1^·d^−1^)	Serum Additive Volume (%)	OD Value	Inhibition Rate (%)
control-FBS	—	—	10	0.9085 ± 0.0070	0
water-control-S	—	—	10	0.9023 ± 0.0073	0
oil-control-S	—	—	10	0.8977 ± 0.0148	0
ADR	0.038	—	10	0.4552 ± 0.0071 **	49.94
5-FU-S	—	0.1000	10	0.4637 ± 0.0034 ^∆∆^	48.56
PEF-S	—	1.1500	10	0.8740 ± 0.0046	2.67
CF-S	—	1.0140	10	0.8662 ± 0.0078	3.56
EAF-S	—	0.9540	10	0.6910 ± 0.0047 ^##^	23.05
BF-S	—	4.6350	10	0.8790 ± 0.0037	2.55
WF-S	—	4.7575	10	0.8902 ± 0.0053	1.33

Note: ** *p* < 0.01 compared with Negative control of fetal bovine serum (control-FBS); ^##^ *p* < 0.01 compared with negative control mice serum containing olive oil (oil-control-S); ^∆∆^ *p* < 0.01 compared with negative control mice serum containing water (water-control-S).

**Table 6:** Effects of mice serum containing fractions of *Sauromatum giganteum* (Engl.) *Cusimano & Hett* Tuber on proliferation of HT-29 cell (*n* = 6).

Groups	Dose (μg·mL^−1^)	Oral Administration Dose (g·kg^−1^·d^−1^)	Serum Additive Volume (%)	OD Value	Inhibition Rate (%)
control-FBS	—	—	10	0.9297 ± 0.0083	0
water-control-S	—	—	10	0.9352 ± 0.0069	0
oil-control-S	—	—	10	0.9315 ± 0.0066	0
ADR	0.082	—	10	0.4633 ± 0.0069 **	50.17
5-FU-S	—	0.1000	10	0.5420 ± 0.0050 ^∆∆^	42.04
PEF-S	—	1.1500	10	0.9115 ± 0.0054	2.15
CF-S	—	1.0140	10	0.9037 ± 0.0087	2.98
EAF-S	—	0.9540	10	0.6072 ± 0.0065 ^##^	34.81
BF-S	—	4.6350	10	0.9127 ± 0.0066	2.41
WF-S	—	4.7575	10	0.9175 ± 0.0047	1.89

Note: ** *p* < 0.01 compared with Negative control of fetal bovine serum (control-FBS); ^##^ *p* < 0.01 compared with negative control mice serum containing olive oil (oil-control-S); ^∆∆^ *p* < 0.01 compared with negative control mice serum containing water (water-control-S).

**Table 7:** Effects of mice serum containing fractions of *Sauromatum giganteum* (Engl.) *Cusimano & Hett* Tuber on proliferation of MDA-MB-231 cell (*n* = 6)

Groups	Dose (μg·mL^−1^)	Oral Administration Dose (g·kg^−1^·d^−1^)	Serum Additive Volume (%)	OD Value	Inhibition Rate (%)
control-FBS	—	—	10	0.9363 ± 0.0307	0
water-control-S	—	—	10	0.9537 ± 0.0191	0
oil-control-S	—	—	10	0.9447 ± 0.0272	0
ADR	0.652	—	10	0.4782 ± 0.0390 **	48.93
5-FU-S	—	0.1000	10	0.7477 ± 0.0351 ^∆∆^	21.59
PEF-S	—	1.1500	10	0.9328 ± 0.0198	1.27
CF-S	—	1.0140	10	0.9303 ± 0.0115	1.59
EAF-S	—	0.9540	10	0.8282 ± 0.0177 ^##^	12.28
BF-S	—	4.6350	10	0.9077 ± 0.0194	4.82
WF-S	—	4.7575	10	0.9203 ± 0.0156	3.56

Note: ** *p* < 0.01 compared with Negative control of fetal bovine serum (control-FBS); ^##^ *p* < 0.01 compared with negative control mice serum containing olive oil (oil-control-S); ^∆∆^ *p* < 0.01 compared with negative control mice serum containing water (water-control-S).

**Table 8:** Effects of different batches of mice serum containing ethyl acetate fraction of *Sauromatum giganteum* (Engl.) *Cusimano & Hett* Tuber on proliferation of tumor cell lines.

Cells	Oral Administration Dose (g·kg^−1^·d^−1^)	Serum Additive volume (%)	Inhibition rate (%)
SMMC-7721	0.9540	10	71.31
SGC-7901	0.9540	10	73.89
MCF-7	0.9540	10	50.75
HeLa	0.9540	10	22.75
A549	0.9540	10	23.05
HT-29	0.9540	10	34.81
MDA-MB-231	0.9540	10	12.28

### 2.2. Antiproliferative Effects of Ethyl Acetate Fractions on 7 Cell Lines

Comparing the data on the inhibition rate of mice serum containing ethyl acetate fraction in Table 1, Table 2, Table 3, Table 4, Table 5, Table 6 and Table 7 (shown in Table 8), we found the inhibition strength of mice serum containing ethyl acetate fraction on different tumor lines is different. However, the mice serum containing ethyl acetate fraction were from different batches of mice, likely to cause errors in results. Therefore, we prepared the mice serum containing ethyl acetate fraction again using the same batch of mice, and observed the cytotoxicity on 7 tumor cell lines by MTT assay. Table 9 shows the result, and we found that the anti-tumor effect shows the same trend as Table 8. The order from strong to weak is SGC-7901 > SMMC-7721 > MCF-7 > HT-29 > A549 > HeLa > MDA-MB-231.

**Table 9:** Effects of mice serum containing ethyl acetate fractions of *Sauromatum giganteum* (Engl.) *Cusimano & Hett* Tuber on proliferation of tumor cell lines (*n* = 6).

Cells	Oral Administration Dose (g·kg^−1^·d^−1^)	Serum Additive Volume (%)	Inhibition Rate (%)
SMMC-7721	0.9540	10	72.13
SGC-7901	0.9540	10	74.33
MCF-7	0.9540	10	50.12
HeLa	0.9540	10	27.40
A549	0.9540	10	28.69
HT-29	0.9540	10	36.75
MDA-MB-231	0.9540	10	14.46

### 2.3. The Result of Acute Toxicity Test

Lethal dose 50 (LD_50_) were found to be >1.150 g/kg b.w. (per os) for the petroleum ether fraction, >1.014 g/kg b.w. (per os) for the chloroform fraction, >0.954 g/kg b.w. (per os) for the ethyl acetate fraction, >4.635 g/kg b.w. (per os) for the *n*-butanol fraction, and >4.7575 g/kg b.w. (per os) for the water fraction in mice. Tested mice of each group did not show any overt signs of toxicity during 24 h and 14 days observation. No mortality was recorded throughout 14 days monitoring.

### 2.4. Effect of Ethyl Acetate Fraction from Sauromatum giganteum *(Engl.)* Cusimano & Hett Tuber on Growth of Transplanted Tumor S_180_ and H_22_ in Mice

We investigated the effect of ethyl acetate fraction on the growth of S_180_ and H_22_ xenograft tumor in mice. After the tumor cells were inoculated, all the mice were inoculated successfully, tumorigenicity was 100%. The tumor weight is shown in Table 10. The results indicated that the growth of implanted sarcoma S_180_ in mice could be significantly inhibited by ethyl acetate fraction of *Sauromatum giganteum* (Engl.) *Cusimano & Hett* tuber in a dosedependent manner compared with the negative control group (*p* < 0.01). The inhibitory rates were 15.22%, 26.15%, and 40.24% at the doses of 106, 318, 954 mg/kg·d Ethyl acetate fraction dry extract, respectively.

**Table 10:** Antitumor activity of ethyl acetate fractions of *Sauromatum giganteum* (Engl.) *Cusimano & Hett* tuber in mice with transplanted S_180_ tumor.

Groups	Doses (mg·kg^−1^·d^−1^)	Weight of Tumor (g)	Inhibitor Rate (%)
Negative control	—	2.13 ± 0.12	0
5-FU	25	0.15 ± 0.01 **	92.91
Ethyl acetate fraction	106	1.80 ± 0.05 **	15.22
	318	1.57 ± 0.05 **^,##^	26.15
	954	1.27 ± 0.08 **^,##,∆∆^	40.24

Note: ** *p* < 0.01 compared with negative control serum; ^##^ *p* < 0.01 compared with 106 mg/kg·d group; ^∆∆^ *p* < 0.01 compared with 318 mg/kg·d group.

Ethyl acetate fraction of *Sauromatum giganteum* (Engl.) *Cusimano & Hett* similarly inhibited the growth of implanted H_22_ hepatoma in mice, leading to a significant tumor regression compared with the control group (*p* < 0.01). The life extending rates were 33.61%, 40.16%, and 55.74% at the doses of 106, 318, 954 mg/kg·d Ethyl acetate fraction dry extract, respectively (Table 11).

**Table 11:** Antitumor activity of ethyl acetate fractions of *Sauromatum giganteum* (Engl.) *Cusimano & Hett* tuber in mice with transplanted H_22_ tumor.

Groups	Doses (mg·kg^−1^·d^−1^)	Survival Time (d)	Life Extending Rate (%)
Negative control	—	12.20 ± 2.20	0
5-FU	25	23.50 ± 1.51 **	92.62
Ethyl acetate fraction	106	16.30 ± 1.57 **	33.61
	318	17.10 ± 1.10 **	40.16
	954	19.00 ± 2.00 **^,#,∆^	55.74

Note: ** *p* < 0.01 compared with negative control serum; ^#^ *p* < 0.05 compared with 106 mg/kg·d group; ^∆^ *p* < 0.05 compared with 318 mg/kg·d group.

### 2.5. UPLC-TOF-MS Analytic Results

**Figure 1:**
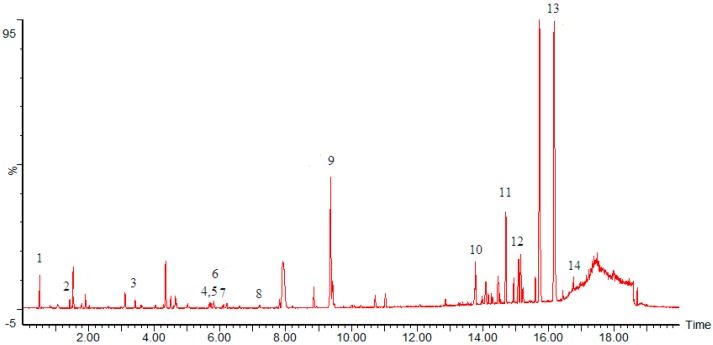
Total current chromatograms of ethyl acetate fraction of *Sauromatum giganteum* (Engl.) *Cusimano & Hett* Tuber in negative mode.

The analysis of active compounds in ethyl acetate fraction of *Sauromatum giganteum* (Engl.) *Cusimano & Hett* Tuber was carried out by UPLC-TOF-MS. Through comparing the t_R_ values and the MS characteristics of the peaks with reference compounds and the literatures, a total of 14 compounds of extracts in ethyl acetate fraction were identified. The 14 identified compounds are listed as follows: sucrose, adenosine, coniferin, lariciresinol, piresil-4-*O*-β-d-glucoside, olivil, piresil-4-*O*-β-d-glucoside (isomer), pinoresinol, tianshic acid, hexadecanedioic acid, linoleic acid, palmitic acid, linolenic acid, linolic acid. The information about the analyzed and identified compounds is summarized in Figure 1 and Table 12.

**Table 12:** UPLC-MS analytic results of ethyl acetate fraction of *Sauromatum giganteum* (Engl.) *Cusimano & Hett* Tuber.

Peak No.	t_R_ (min)	*m/z*	Molecular Formula	Identification
1	0.57	341.1094	C_12_H_22_O_11_	sucrose
2	1.79	312.0948	C_10_H_13_N_5_O_4_	adenosine
3	3.63	387.1306	C_16_H_22_O_8_	coniferin
4	5.68	359.1514	C_20_H_24_O_6_	lariciresinol
5	5.68	565.1923	C_26_H_32_O_11_	piresil-4-*O*-β-d-glucoside
6	5.76	375.1451	C_20_H_24_O_7_	olivil
7	6.20	519.1875	C_26_H_32_O_11_	piresil-4-*O*-β-d-glucoside (isomer)
8	7.36	357.1347	C_20_H_22_O_6_	pinoresinol
9	9.37	329.2326	C_18_H_34_O_5_	tianshic acid
10	13.75	285.2079	C_16_H_30_O_4_	hexadecanedioic acid
11	14.71	279.2333	C_18_H_32_O_2_	linoleic acid
12	15.16	255.2335	C_16_H_32_O_2_	palmitic acid
13	16.21	277.2176	C_18_H_30_O_2_	linolenic acid
14	16.76	279.2336	C_18_H_32_O_2_	linolic acid

## 3. Experimental Section

### 3.1. Materials

The tuber of *Sauromatum giganteum* (Engl.) *Cusimano & Hett* Tuber were purchased from the Harbin Pharmaceutical Group (Harbin, China). The field studies did not involve endangered or protected species. The study protocol was approved by Animal Ethics Committee, Harbin Commerce University. RPMI 1640 culture medium was purchased from GIBCO BRL (Gaithersburg, MD, USA). Fetal bovine serum (FBS) was purchased from Hyclone company (Logan, UT, USA). 3-(4,5-dimethylthiazol-2-yl)-2,5-diphenyl-tetrazolium bromide (MTT) and fluorouracil (5-FU) were purchased from Sigma Chemical, Co. Ltd. (St. Louis, MO, USA). Adriamycin (ADR) were purchased from Pfizer Inc. (New York, NY, USA).

### 3.2. Preparation of Fractions from Sauromatum giganteum *(Engl.)* Cusimano & Hett

*Sauromatum giganteum* (Engl.) *Cusimano & Hett* Tuber was purchased from Harbin Pharmaceutical Group (Harbin, China) and identified as the tuber part of *Sauromatum giganteum* (Engl.) *Cusimano & Hett* by D.-L. Zhang (The School of Pharmacy, Harbin Commerce University, Harbin, China).

**Figure 2:**
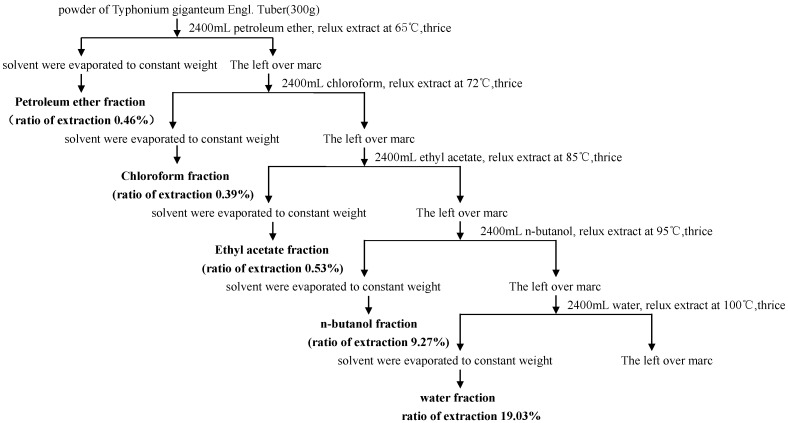
Preparation of fractions from *Sauromatum giganteum* (Engl.) *Cusimano & Hett*.

A separate extraction was performed on the dried plant material to obtain various solvent fractions following the method described by Tadiwos Feyissa *et al.* [28], with slight modifications. Thus, powdered air-dried tuber (300 g) of *Sauromatum giganteum* (Engl.) *Cusimano & Hett* were successively extracted with petroleum ether. The leftover marc was then extracted with chloroform, ethyl acetate, *n*-butanol and distilled water by turning the maceration under reflux to obtain the respective fractions (show in Figure 2). The petroleum ether fraction, chloroform fraction, ethyl acetate fraction, *n*-butanol fraction and water fraction solvent were evaporated in a rotary vacuum evaporator at 40 °C to a constant weight of dry extract. The extraction ratio of petroleum ether fraction, chloroform fraction, ethyl acetate fraction, *n*-butanol fraction and water fraction was 0.46%, 0.39%, 0.53%, 9.27% and 19.03%, respectively. The obtained extracts were kept in air-tight containers wrapped with aluminum foil and stored in a refrigerator until use.

Ai FW and Ma YL *et al.* [12,29] reports 16 compounds were isolated and identified by ESI-MS and NMR from *Sauromatum giganteum* (Engl.) *Cusimano & Hett* Tuber. coniferin (1), 5-hydroxy methyl-2-furaldehyde (2), pinoresinol-4-*O*-β-d-glucopyranoside (3), pinoresinol (4), neoolivil (5), lariciresinol (6), methylconiferin (7), β-sitosterol (8), tianshic acid (9), palmitic acid (10), cinnamic acid (11), and daucosterol (12) were from the ethyl acetate fraction; uridine (13), and adenosine (14) were from the *n*-butanol fraction; 1-decanoyl-rac-glycerol (15), and 3-Glyceryl monooleate (16) were from the petroleum ether fraction.

### 3.3. Animals

Kunming mice of both sexes were used in this study, which were purchased from Changchun Gao-Xin Experimental Animal Center (Changchun, China) with an initial body weight of 18–22 g.

The animals were given water and food *ad libitum*. The temperature of the animal laboratory was controlled within 20 ± 2 °C, the humidity was 50%–60% with a natural day-night cycle. All animal care and treatments were carried out in accordance with the recommendations of the Guide for the Care and Use of Laboratory Animals published by the National Institute of Health, U.S.A.

After 3 days of acclimation. Eighty mice were randomly divided into eight groups with ten mice per group (each group with five male mice and five female mice): water control group, oil control group, positive control group (5-FU), petroleum ether fraction group, chloroform fraction group, ethyl acetate fraction group, *n*-butanol fraction group, and water fraction group.

### 3.4. Administration of Fractions from Sauromatum giganteum *(Engl.)* Cusimano & Hett

The petroleum ether fraction, chloroform fraction, and ethyl acetate fraction were respectively dissolved in oil, but the *n*-butanol fraction and water fraction were respectively dissolved in distilled water before administration at saturation.

The animals were fasted for 12 h, but given water ad libitum before administration. The treatment groups of animals (*n* = 10) were orally administered different fractions, twice a day. The animals the in water control group and oil control group were orally administered distilled water and olive oil respectively, The animals in the positive control group were orally administered 100 mg/kg·d 5-FU, and they were run concurrently with different fraction-treated groups. The animals in the petroleum ether fraction group, chloroform fraction group, ethyl acetate fraction group, *n*-butanol fraction group, and water fraction group were orally administered at saturation concentrations of 1.150, 1.014, 0.954, 4.635 and 4.7575 g/kg·d in a final volume 0.5 mL. The animals were fasted for 12 h but given water *ad libitum* before collection of serum.

### 3.5. Preparation of Fraction-Containing Serum

Sixty minutes after the seventh treatment, the blood of the animals was extracted by eyeball extirpating one by one under aseptic condition, and let stand at 4 °C for twelve hours, then centrifuged at 3000× *g* for 15 min. Both fraction-containing serum and control serum were filtered through a 0.22 μm micropore film (Millipore, Danvers, MA, USA), termed as PEF-S (petroleum ether fraction-containing mice serum), CF-S (chloroform fraction-containing mice serum), EAF-S (ethyl acetate fraction-containing mice serum), BF-S (*n*-butanol fraction-containing mice serum), WF-S (water fraction-containing mice serum), 5-FU-S (5-FU-containing mice serum), water-control-S (Negative control of serum containing water) and oil-control-S (Negative control of mice serum containing olive oil) and control-FB-S (Negative control of fetal bovine mice serum), respectively, and stored at −80 °C until use [30].

### 3.6. Cell Culture

The human SMMC-7721 hepatocellular carcinoma, human SGC-7901 gastric carcinoma, human MCF-7 breast adenocarcinoma, human HeLa cervix adenocarcinoma, human A549 lung carcinoma epithelial, human HT-29 colon adenocarcinoma, and human MDA-MB-231 breast adenocarcinoma cell lines were obtained from Harbin Medicine University (Harbin, China). Cells were cultured in RPMI 1640 medium (Gibco, 31800-022) supplemented with 10% (v/v) fetal bovine serum (Gibco, 10099-141), 100 U/mL penicillin, 100 μg/mL streptomycin and 1 mM L-glutamine at 37 °C in an atmosphere of 5% CO_2_. The medium was renewed two or three times/week. Cells in the logarithmic growth phase were used for further experiments.

### 3.7. Cytotoxicity Activity of Fraction-Containing Serum on the Human Tumor Cell Lines

The cytotoxicity of 5 different fraction-containing serum on the human tumor cell lines were evaluated using MTT assay. The cells treated with fractions-containing serum were incubated for 72 h after which the MTT [3-(4,5-dimethylthiazole-2-yl)-2,5-diphenyltetrazolium bromide] assay was carried out as described by Mahmoud, *et al.* [31,32], but with slight modifications. Cells were plated in 96-well plates (4 × 10^3^ cells/well for SMMC-7721 cells, 8 × 10^3^ cells/well for SGC-7901 cells, 6 × 10^3^ cells/well for MCF-7 cells, 8 × 10^3^ cells/well for HeLa cells, 1 × 10^4^ cells/well for A549 cells, 8 × 10^3^ cells/well for HT-29 cells, 8 × 10^3^ cells/well for MDA-MB-231 cells) in 100 µL of RPMI 1640 medium containing 10% (v/v) fetal bovine serum for 24 h incubation. After 24 h, the cells were cultured for 72 h in RPMI 1640 medium containing 10% serum of rats treated with different fractions of *Sauromatum giganteum* (Engl.) *Cusimano & Hett* Tuber. Adriamycin (ADR), which is widely used for the treatment of several human cancers, was used as the positive reference as well as 5-FU-S in this study. At the end of 72 h incubation, the medium were discarded and 100 μL of MTT stock solution (1 mg/mL) were added to each well and the plates were further incubated. Four hours later, DMSO (150 μL) was added to each well to solubilize the water-insoluble purple formazan crystals. The amount of MTT-formazan is directly proportional to the number of living cells and was determined by measuring the optical density (OD) at 570 nm using microplate reader (Bio-Rad). The percentage of cytotoxic activity compared to the untreated cells was determined using the following equation [33,34]:

Cell inhibitory rate (%) = (OD of control cells − OD of treated cells) × 100/OD of control cells

### 3.8. Cytotoxicity Activity of Ethyl Acetate Fraction-Containing Serum on 7 Human Tumor Cell Lines

The assay and operation was same with 3.7, ethyl acetate fraction is an effective anti-tumor bioactive solvent fraction according to our experimental data in 3.7 (Table 8). However, the mice serum containing ethyl acetate fraction were from different batches of mice, likely to cause errors in results. Therefore, using the same batch of mice, we prepared the mice serum containing ethyl acetate fraction again and observed the cytotoxicity on SMMC-7721, SGC-7901, MCF-7, HT-29, HeLa, A549, MDA-MB-231 cell by MTT assay (Table 9).

### 3.9. Acute Toxicity Test

Fifty normal kunming mice (20 ± 2 g) of either sex, fed with pellet diets were randomly divided into five groups with ten mice per group (each group with five male mice and five female mice) and administrated with 1.150 g/kg of the petroleum ether fraction, 1.014 g/kg of the chloroform fraction, 0.954 g/kg of the ethyl acetate fraction, 4.635 g/kg of the *n*-butanol fraction, 4.7575 g/kg of the water fraction orally by gastric gavage. The animals were observed for general behavioral changes, signs of toxicity and mortality continuously for 1 h after treatment, then intermittently for 4 h, and thereafter over a period of 24 h. Further, the mice were observed for up to 14 days following the treatment for any lethality and death [35].

### 3.10. In Vivo Antitumor Effect of Ethyl Acetate Fraction on Mice Bearing-S_180_ or H_22_ Cell Line

S_180_ or H_22_ tumor cells were harvested and washed three times with normal saline (NS). The cells were pelleted by brief centrifugation at 300× g. The supernatant was aspirated, and the cells were resuspended in NS at a density of 1 × 10^7^ cells/mL. The mice were subcutaneously implanted with 2 × 10^6^ cells/mouse on the right oxter for S_180_-tumor-model or peritoneum for H_22_-tumor-model (day 0). Twenty-four hours after inoculation, fifty mice with S_180_ cells or fifty mice with H_22_ cells were randomly divided into 5 groups, respectively.

After tumor implantation for 24 h, mice were administered orally with various doses of ethyl acetate fraction of *Sauromatum giganteum* (Engl.) *Cusimano & Hett* Tuber (dissolved in oil, twice a day, 106, 318, 954 mg/kg·d Ethyl acetate fraction dry extract) for 7 days. 5-fluorouracil (25 mg/kg·d) was served as a positive drug.

24 h after the last administration, the mice bearing S_180_ tumor lines were sacrificed and the tumors were excised and weighted. The inhibition rate (IR) of tumor growth was calculated by the following formula: IR (%) = [(average tumor weight of the control group − average tumor weight of the treatment group)/average tumor weight of the control group] × 100.

The mice bearing H_22_ tumor lines were recorded the survival time, the life span were determined using the following equations: increase in life span (%) = [(median survival time of treated mice − median survival time of untreated mice)/median survival time of untreated mice] × 100.

All experimental procedures were performed in accordance with the Guide lines of Animal Experiments from the Committee of Medical Ethics, National Health Department of China (1998).

### 3.11. Analytical Conditions of Ethyl Acetate Fraction of Sauromatum giganteum *(Engl.)* Cusimano & Hett by UPLC- TOF-MS

The chromatographic separation was achieved on Waters Acquity UPLC HSS T3 (2.1 mm × 100 mm, 1.8 μm) column by employing the Waters Acquity UPLC system (Waters Corp., Milford, MA, USA) consisting of a binary solvent manager, a sample manager and a column temperature controller. The mobile phase which consisted of 0.1% formic acid aqueous solution (A)-0.1% formic acid Acetonitrile solution (B) using a gradient elution (Table 13), was pumped at a flow rate of 0.5 mL·min^−1^. The temperatures of the column and sample manager were maintained at 40 °C and 4 °C, respectively.

Waters SYNAPT G2-S Mass Spectrometers (Waters Corp., Milford, MA, USA) equipped with an electrospray ionization (ESI) source was employed for detection. The mass spectrometry was operated in negative ionization mode. In order to achieve maximum sensitivity, the mass spectrometric conditions were optimized as follows:cone gas (nitrogen) flow rate, 10 L/h; desolvation gas (nitrogen) flow rate, 800 L/h; capillary voltage, 2500 V; source temperature, 120 °C; desolvation temperature, 500 °C; scan range: *m/z* 50–1500. Data processing was performed with UNIFI 1.7 software.

**Table 13:** Mobile phase gradient elution system.

Time (min)	A (%)	B (%)	flow-Rate (mL/min)
0	99	1	0.5
0.3	99	1	0.5
6	75	25	0.5
9	60	40	0.5
12	50	50	0.5
17	1	99	0.5
18	1	99	0.5
20	99	1	0.5

### 3.12. Statistical Analysis

The data is represented as the mean ± SD. Statistical significance was calculated using student’s *t*-test. *p*-values of 5% or less were considered statistically significant.

## 4. Conclusions

Five different fraction-containing serums (petroleum ether fraction, chloroform fraction, ethyl acetate fraction, n-butanol fraction and water fraction of *Sauromatum giganteum* (Engl.) *Cusimano & Hett*) were prepared and investigated for the inhibition activity on tumor cells, respectively. Only the mice serum containing ethyl acetate fraction of *Sauromatum giganteum* (Engl.) *Cusimano & Hett* Tuber could inhibit the tumor cells proliferation. The inhibition effection of ethyl acetate fraction on the seven cell lines in descending order is SGC-7901 > SMMC-7721 > MCF-7 > HT-29 > A549 > HeLa > MDA-MB-231. Sarcoma S_180_ and hepatoma H_22_ tumor-bearing mouse models are adopted in antitumor experiment *in vivo* [36,37,38,39], We found ethyl acetate fraction of *Sauromatum giganteum* (Engl.) *Cusimano & Hett* Tuber could inhibit the S_180_ growth and prolonged the life span of mice bearing H_22_ with the dosage increase.

Therefore, Ethyl acetate fraction of *Sauromatum giganteum* (Engl.) *Cusimano & Hett* is the anti-tumor activity fraction. Anti-tumor activity on cancer cell line in descending order is SGC-7901 > SMMC-7721 > MCF-7 > HT-29 > A549 > HeLa > MDA-MB-231.
